# Combined Metabolome and Lipidome Analyses for In-Depth Characterization of Lipid Accumulation in the DHA Producing *Aurantiochytrium* sp. T66

**DOI:** 10.3390/metabo11030135

**Published:** 2021-02-25

**Authors:** Zdenka Bartosova, Helga Ertesvåg, Eirin Lishaugen Nyfløt, Kristoffer Kämpe, Inga Marie Aasen, Per Bruheim

**Affiliations:** 1 Department of Biotechnology and Food Science, NTNU Norwegian University of Science and Technology, 7491 Trondheim, Norway; Zdenka.Bartosova@ntnu.no (Z.B.); Helga.Ertesvag@ntnu.no (H.E.); eirin_nyf@hotmail.com (E.L.N.); kampekristoffer@gmail.com (K.K.); 2 Biotechnology and Nanomedicine, SINTEF Industry, 4730 Trondheim, Norway; Inga.M.Aasen@sintef.no

**Keywords:** thraustochytrids, DHA, metabolomics, lipidomics, metabolic engineering, mass spectrometry, chromatography

## Abstract

Thraustochytrids are marine heterotrophic microorganisms known for their potential to accumulate docosahexaenoic acid (DHA)-enriched lipids. There have been many attempts to improve thraustochytrid DHA bioprocesses, especially through traditional optimization of cultivation and media conditions. Nevertheless, thraustochytrid-based bioprocesses are still not commercially competitive for high volume-low cost production of DHA. Thus, it is realized that genetic and metabolic engineering strategies are needed for the development of commercially competitive thraustochytrid DHA cell factories. Here, we present an analytical workflow for high resolution phenotyping at metabolite and lipid levels to generate deeper insight into the thraustochytrid physiology, with particular focus on central carbon and redox metabolism. We use time-series sampling during unlimited growth and nitrogen depleted triggering of DHA synthesis and lipid accumulation (LA) to show-case our methodology. The mass spectrometric absolute quantitative metabolite profiling covered glycolytic, pentose phosphate pathway (PPP) and tricarboxylic acid cycle (TCA) metabolites, amino acids, complete (deoxy)nucleoside phosphate pools, CoA and NAD metabolites, while semiquantitative high-resolution supercritical fluid chromatography MS/MS was applied for the lipid profiling. Interestingly, trace amounts of a triacylglycerols (TG) with DHA incorporated in all three acyl positions was detected, while TGs 16:0_16:0_22:6 and 16:0_22:6_22:6 were among the dominant lipid species. The metabolite profiling data indicated that lipid accumulation is not limited by availability of the acyl chain carbon precursor acetyl-CoA nor reducing power (NADPH) but rather points to the TG head group precursor glycerol-3-phosphate as the potential cause at the metabolite level for the gradual decline in lipid production throughout the cultivation. This high-resolution phenotyping provides new knowledge of changes in the central metabolism during growth and LA in thraustochytrids and will guide target selection for metabolic engineering needed for further improvements of this DHA cell factory.

## 1. Introduction

Thraustochytrids are interesting microorganisms, both from biological and industrial points of view [1,2,3,4]. These unicellular, mainly marine microorganisms are able to synthesize large quantities of the long-chain omega-3 fatty acid docosahexaenoic acid (DHA) and store it as tri-acylglycerols in their intracellular lipid bodies. There are studies reporting more than 50% lipids in the final biomass and with over 25% of DHA of the total lipid pool (reviewed in [5]). Thraustochytrids have long been used to produce DHA for the high-end healthcare market. Due to the depletion of main cheap sources as fish oil for long chain poly-unsaturated fatty acids (PUFA), especially DHA, but also eicosapentaenoic acid (EPA), there is now opening up of a low cost market for microbial production of PUFA, e.g., salmon farming is a large consumer of DHA-rich oils, and oil from thraustochytrids are already being used for this purpose.

Several thraustochytrid species have been genome sequenced, and the information including deep manual annotation has been released to the community [6,7,8]. Further, several transcriptome studies have provided time-series information about transitions in gene expression during growth and lipid accumulation [6,9,10]. From a bioprocess point of view this opens larger opportunities to manipulate the cells for both increased production rates and relative increase of DHA content. 

**Figure 1:**
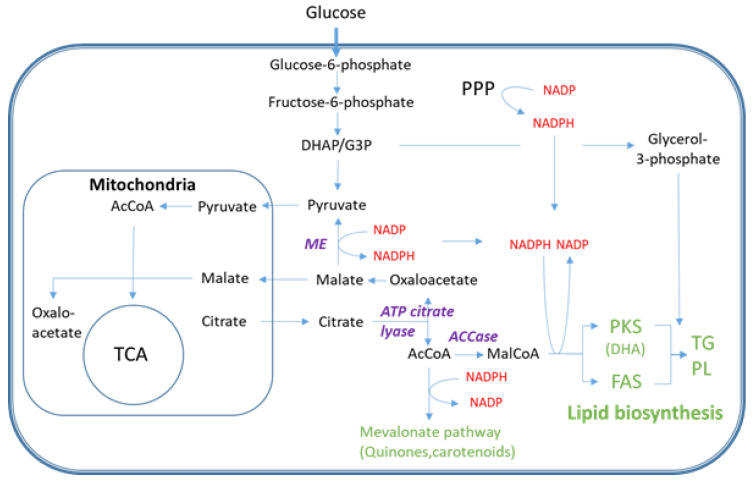
Overview of the metabolic pathway for DHA (PKS) and saturated fatty acid (FAS) biosynthesis with focus on carbon precursor and NADPH formation. Abbreviations not explained in the text: AcCoA: acetyl-CoA, MalCoA: malonyl-CoA, ACCase: AcCoA carboxylase, DHAP: dihydroxyacetone phosphate, G3P: glyceraldehyde 3-phosphate, PPP: pentose phosphate pathway, TCA: tricarboxylic acid cycle, PL: phospholipid.

Thraustochytrids mostly use polyketide synthase (PKS) enzyme systems for DHA biosynthesis, while shorter chain fatty acids (e.g., C16 and C18) are synthetized by the common fatty acid synthesis (FAS) pathway [11,12,13]. Figure 1 present key pathways, branch points and metabolites of critical importance for lipid biosynthesis in thraustochytrids. Acetyl-CoA and malonyl-CoA are precursors of fatty acid synthesis and common for the PKS and FAS pathways. A gene encoding ATP citrate lyase (ACL), which is the primary enzyme for synthesis of cytosolic acetyl-CoA, has been found in some, but not all sequenced thraustochytrid genomes [6]. The reducing power NADPH can be produced by two enzymatic steps in pentose phosphate pathway (PPP), iso-citrate dehydrogenase in tricarboxylic acid (TCA) cycle and by malic enzyme (decarboxylating malate dehydrogenase) which shunts carbon from TCA (malate) to lower glycolytic pathway (pyruvate) as an anaplerotic reaction, although other pathways cannot be excluded [14]. During the last decade there have been many efforts to improve productivities and yields of DHA production and lipid accumulation with traditional bioprocess means [15,16,17,18,19,20,21]. That includes both optimization of medium composition and bioreactor conditions [22]. The highest productivities have been obtained using rich media with high concentrations of yeast extract and/or peptones. Still, the specific productivity (g DHA/g cell/hour) in industrial scale fermentations need to be improved to reduce the production costs for competitive entry on the low-cost market. Thus, new means need to be used which implies metabolic engineering (ME) strategies [23]. Establishment of genetic tools for thraustochytrid strain engineering has been challenging, but several successful attempts have been reported [24,25,26,27,28]. Most thraustochytrid studies are reported with cultivation data, total lipids and DHA-production, while only scarce information is available about intracellular metabolite levels [13,29,30,31,32]. Such information is requested in ME projects, both to generate biological knowledge as basis for selection of targets to manipulate, but, equally important, to characterize the phenotype of the resulting mutants. In this report we present a workflow for comprehensive metabolic profiling of central carbon and energy metabolism of thraustochytrids that provide new and essential knowledge for further developments of these microorganisms as industrial DHA production strains. Standard analytical protocols needed adaptation to this model system, since there is a large biomass increase during the time series sampling, and also, as important, the biomass composition changes dramatically. This poses challenges on the analytical methodology and downstream processing of the quantitative data, and we describe the development and adjustment of a portfolio of quantitative mass spectrometry-based metabolite profiling methods and high-resolution lipid profiling for characterization of DHA incorporation into individual lipid species. We demonstrate the analytical approach by using as basis a time-series study during growth and nitrogen depletion triggering of DHA synthesis and lipid accumulation (LA). 

## 2. Results

### 2.1. Cell Growth and Lipid Accumulation

Metabolome time-series sampling requires 20–50 mL per time point; thus, such studies cannot be performed in shake flasks, but must be run in bench-top bioreactors with at least 1–2 L cultivation volume. Additional benefits by using bioreactors are continuous monitoring and controlling of pH and oxygenation. The medium designed for the current study is dilute compared to industrial media designed to reach high cell densities. We also chose to use a mineral medium with glucose as only carbon and energy source and ammonium as nitrogen source. This is a poorer condition than productive industrial conditions, but is a simpler system for high quality metabolome sampling and sample processing and calculation of biomass and lipid yields on carbon source. The protocols will be adjusted to and validated for industrially closer conditions in future work.

**Figure 2:**
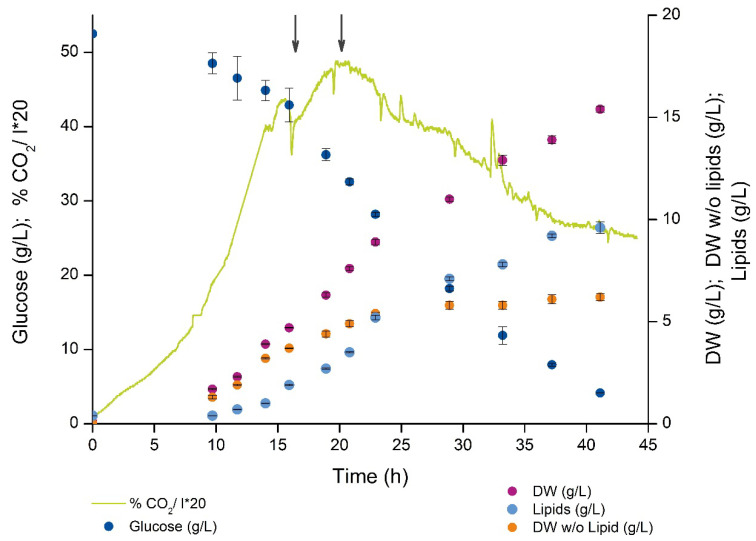
Time-course profile for the nitrogen limited *Aurantiochytrium* sp. T66 bioreactor cultivation showing the continuous CO_2_ exhaust gas production (left *y*-axis, percentage in off-gas*20), glucose consumption (left *y*-axis, in g/L) and biomass formation (with and without intracellular lipids), and total lipid (TL) formation (biomass and lipid concentration in grams/L on the right *y*-axis). The two arrows indicate metabolic events discussed in the text. One representative biological replica is shown, error bars indicate standard deviation of at least three resamplings.

The present medium supported almost 5 g dry weight (DW)/L before nitrogen was exhausted from the medium around 16 h as indicated by the sharp transient drop in the CO_2_ production (Figure 2, left arrow). The lipid free biomass was leveling off, while the intracellular accumulation of lipids lasted until the end of the fermentation. Lipid synthesis rate was not constant but highest in the first part of the cultivation (until 20–25 h) and decreased in the last period (25–45 h). Compared to non-oleaginous microorganisms, the cultivation showed an unusual CO_2_ profile after the nitrogen depletion, since there was only a low 15–20% drop in CO_2_ and rebuilding to a new maximum 5 h after depletion (around 20 h, right arrow in Figure 2). Around 30% of the glucose was converted to biomass in the growth phase while glucose conversion to lipid in LA phase was in the 20–30% range (Y_XS_ and Y_PS_ can be found in Supplementary Table S1, respectively, on gram/gram basis) which is close to the theoretical maximal yield estimated to be around 0.3 g DHA/g glucose [1]. The rest of the glucose carbon was oxidized and released as CO_2_, no extracellular accumulation of short or medium chain organic acids was observed in neither of the cultivation phases.

### 2.2. Fatty Acid and Lipid Profiling

**Figure 3:**
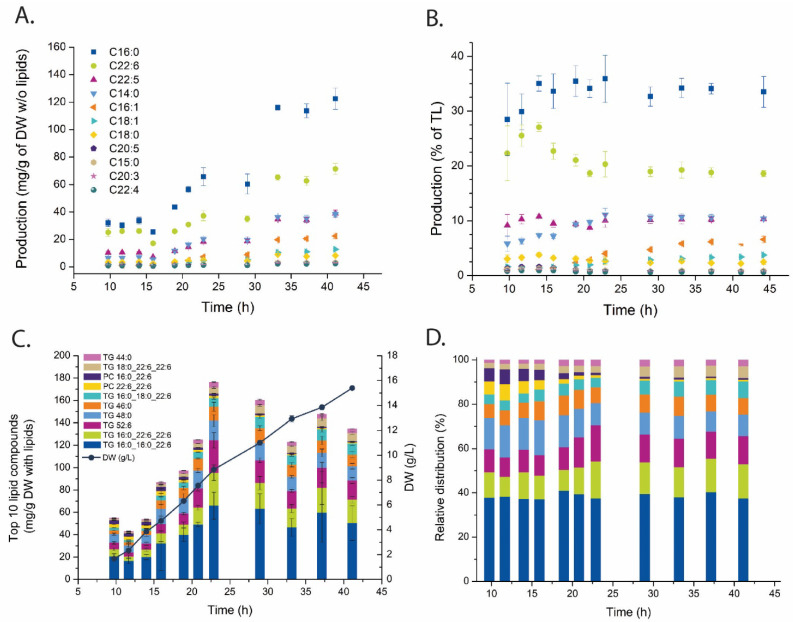
Fatty acid and lipid time series profiling. (**A**,**B**) Concentration profile and relative distribution of fatty acids, and (**C**,**D**) the 10 most abundant lipid species. (**A** and **B**), (**C** and **D**) share same legends, respectively. TG—triglycerides, PC—phosphatidylcholines. Tabulated data and full identities can be found in Supplementary Tables S2 and S3.

The fatty acid composition of the lipid fractions was first determined to describe the production of polyunsaturated vs. saturated fatty acids, i.e., PKS vs. FAS produced (see Figure 1). This is the standard analysis of DHA content in microbial lipid accumulation systems and the results reproduce earlier reports on thraustochytrids. The dominating fatty acids were palmitic acid (C16:0) and DHA (C22:6) (Figure 3A), 30% and 20% of the total fatty acid pool, respectively (Figure 3B). 

Lipid analysis was performed using supercritical fluid chromatography (SFC) coupled with high resolution QTOF mass spectrometer. Since the SFC separates the lipids based on their polarity with base line separation of lipid classes both database-identified and -unidentified lipid species could be included in the downstream data analysis [33]. Further, the inclusion of internal and external standards enabled a semiquantitative inspection of the results. This is important for the interpretation since there is high variation in response among the various lipid species. Eight out of the 10 most abundant lipid compounds were TGs, and these contained mostly palmitic and docosahexaenoic fatty acyls (Figure 3C). These 10 most abundant lipid compounds represented almost 40% of the total lipid abundance at the start of the cultivation and decreased to 25–30%, and half of this fraction was made up to the two most abundant lipids–TG 16:0_16:0_22:6 and TG 16:0_22:6_22:6 (Figure 3D). A TG with DHA in all three acyl positions was also detected, but did not rank among the 10 most abundant.

### 2.3. Metabolite Profiling

Absolute quantitative metabolite profiling was performed with six MS based methods using different chromatographic separation. The metabolome sampling was performed at every second sampling point (six out of 11) due to the extensive workload from sampling, sample processing, and LC-MS analysis [34]. The amino acids and organic acids were derivatized prior to analysis, while the other metabolite classes could be analyzed without derivatization. Sampling and sample processing were also optimized to the individual MS-methods to achieve optimal chromatography for highest quantitative accuracy and precision. Some metabolites are highly unstable, i.e., CoAs and reduced NADs, and those samples needed dedicated sampling and sample processing protocols and immediate analyzing directly after extraction (see the comments in the extra paragraph in Section 4 for sampling and MS analyses of these metabolites). All analyses were performed with ^13^C internal standard dilution strategy [35] and results can be converted to μmol/g DW (lipid free); thus, results from all six methods can be merged into one table (Supplementary Table S4). Relative standard deviation was in the 10–30% range for most metabolites, some highly unstable and low abundant metabolites (e.g., NADPH and SucCoA) were more challenging analytes and higher variation among technical replicas must be accepted for such analytes. The technical variation is higher than the biological as for most microbial model systems, and we chose to present one biological replica since there are off-sets both along time and concentration axis among individual cultivations and it is not straight forward how to merge data from different biological replicas.

**Figure 4:**
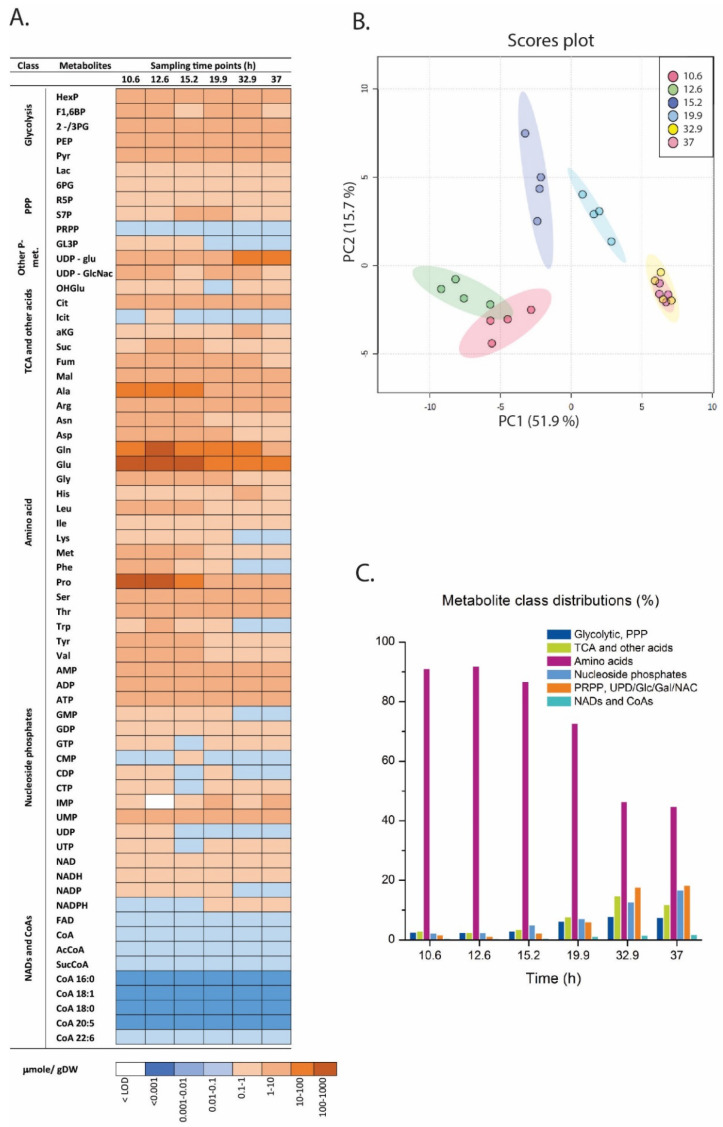
Intracellular metabolite profiling using the six MS based methods for six sampling points (10.6 and 12.6 h—growth phase; 15.2 and 19.9 h—transition phase; 32.9 and 37.0—lipid accumulation (LA) phase). (**A**) Absolute concentrations in µmol/g DW of all quantified metabolites. Average of four technical replicas from one representative biological replica (i.e., cultivation) is presented. Long chain CoAs were obtained from a separate cultivation and are aligned along the time axis for incorporation in the same plot (bar plot of the same data set is presented in Supplementary Figure S1). (**B**) Principal component analysis of the metabolome time series with six sampling points. The score plot displays analysis without normalization but autoscaled. (**C**) Relative metabolite class distributions during the cultivation.

The metabolite concentrations varied over 5–6 orders of magnitude (Figure 4A) and was dominated by a few amino acids (Ala, Glu, Gln, Pro). Glutamate stands out and contributed 30–40% of the whole metabolite pool (Supplementary Figure S1). Proline, which constituted 20% of the central metabolome pool in the growth phase, was drastically reduced in the LA phase. Principal component analysis returned a distinct time-series clustering (Figure 4B). The growth phase samples (1 and 2) clustered together on the left of PC1 while the LA phase samples (5 and 6) were clustering to the right. The two samples in the transition phase (3 and 4) were placed in-between. However, this PCA result is highly biased by concentration differences. The data is already normalized to g DW which is a valid unit for time-series evaluation, but a large reduction in total metabolite pools was observed, up to 80% reduction was observed in LA phase vs. the growth phase. The most obvious reason is that the intracellular cytoplasmic volume became occupied with lipid droplets which reduces the free cytoplasmatic space. However, re-running PCA using normalized to sum returned the same time-series clustering, which indicates that the metabolome data set contains distinct metabolite profiles throughout the cultivation phases. This is clearly seen at the overall level when inspecting the relative metabolite class distributions where the total amino acid pool is decreasing from >90% in the growth phase to <50% in the LA phase while all other classes are increasing (Figure 4C). These are considerations at the relative level, while the correct phrasing at the absolute concentration level is that the decrease in amino acid pools was much higher than for the other metabolite classes.

**Figure 5:**
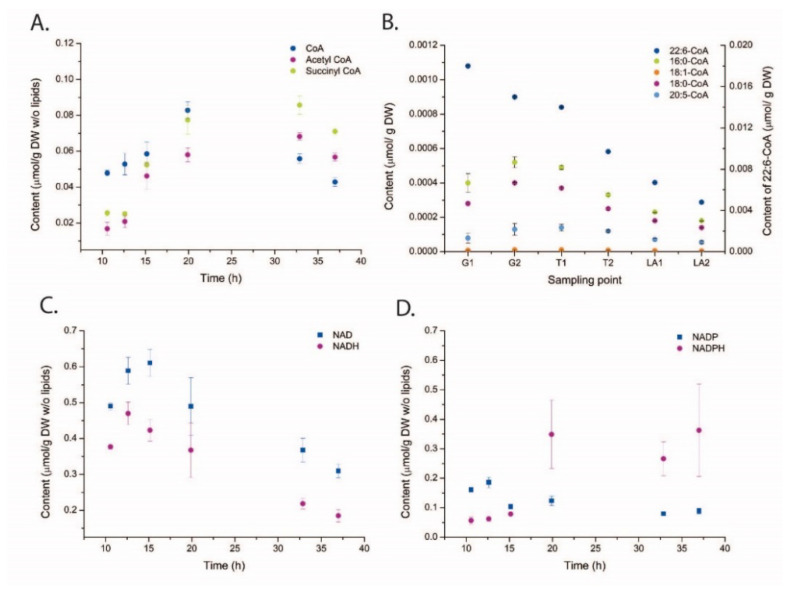
Time series absolute concentrations in µmol/g DW units of SC-CoAs (**A**), LC-CoAs (**B**), NAD (H) (**C**), and NADP(H) (**D**). Metabolites are presented. The data are presented with averaged and standard deviation among four technical replicas. LC-CoA samples are from another biological replica and the samples are aligned to cultivation phases along the time axis (G—growth; T—transition; LA—lipid accumulation).

The absolute concentration heatmap in Figure 4A with decade concentration division gives a coarse impression of the development of individual metabolites from the growth phase through the transition into the LA phase, and several important metabolites with low levels of absolute concentrations are presented in Figure 5. Fatty acid and lipid syntheses are dependent on acetyl-CoA and malonyl-CoA (short chain SC-CoAs) as precursor metabolites and NADPH as reducing cofactor (Figure 1). Malonyl-CoA levels were below LOD (limit of detection) for all sampling points, but interestingly the absolute acetyl-CoA concentration was increasing after growth ceased and remained high also at the last sampling point (Figure 5A). However, the long chain (LC)-CoAs were all slightly decreasing throughout the cultivation and with a remarkable 10 times higher concentration of DHA-CoA vs. the FAS generated acyl-CoAs (Figure 5B). Furthermore, a steady decline in NAD and NADH levels were observed (Figure 5C) while the NAPDH levels were actually increasing at the end of the cultivation (Figure 5D). 

**Figure 6:**
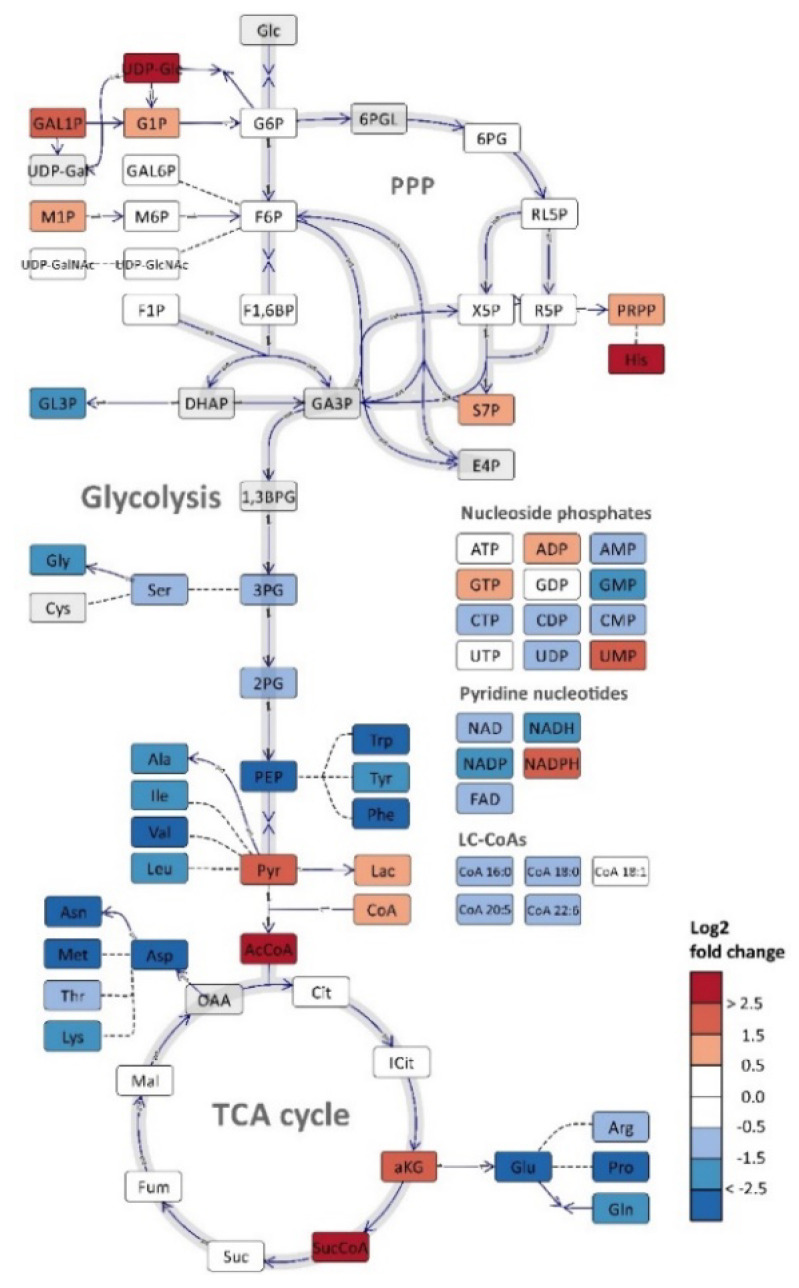
Log_2_ ratio of metabolome data of sampling points 32.9 (LA phase) and 12.6 (growth phase) from the *Aurantiochytrium* sp. T66 cultivation. The figure was prepared using Omix visualization software [38], and data normalized to free available intracellular volume was used. Compartmentalization of eukaryotic cells is not included. Reactions with two opposite arrows indicate reversible reactions with different enzymes, bidirectional arrows are reversible reactions, dashed lines indicate that intermediates are not included.

Next, and to summarize the metabolite profiling, the log_2_ ratio between a LA phase time point and a growth phase time point is presented (Figure 6). The ratios are based on normalization to free cytoplasmatic volume as we judge this to be the most correct normalization strategy in the absence of size and intracellular volume measurements. Free cytoplasmatic volume was calculated by assuming the same cell size in all cultivation phases and that the intracellular lipid droplets displaced volume equivalent to lipid volume increase (see comments on this approach in Section 3). The levels of glycolytic metabolites were relatively unchanged, even though the glucose uptake rate decreased with 60–80% in the LA phase (Figure 6). Some sugar phosphates related to cell wall biosynthesis (UPD-Glu/Gal) accumulated after growth cessation, as did the nucleoside precursor PRPP. Furthermore, almost all amino acids pools showed a dramatic decrease, while the TCA metabolites were unchanged except one, α-KG. This TCA metabolite is important for the nitrogen metabolism in both prokaryotic and eukaryotic cells and are known to accumulate during N-depletion [36,37]. Finally, it must be mentioned again that the pools of acetyl-CoA and NADPH that are necessary for fatty acid synthesis increased in LA phase, while DHA-CoA (and the other LC-CoAs as well) and the lipid head group precursor glycerol-3P decreased, 40% and 70% respectively.

## 3. Discussion

**Figure 7:**
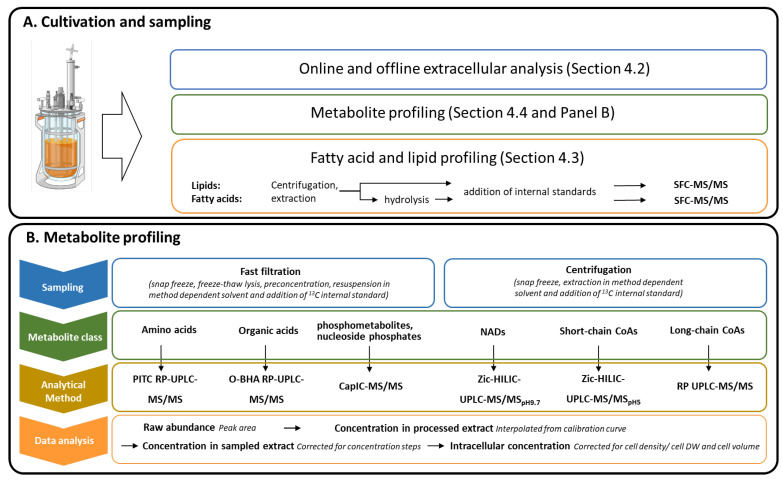
Three different sampling flows were removed from the 2-L bioreactor at appropriate time points to cover growth, transition and LA phases (**A**). A more detailed overview of the six methods metabolite profiling approaches (**B**). The bioreactor illustration was generated using a biorender template (biorender.com).

In this paper we presented a time series monitoring at metabolite and lipid levels of an *Aurantiochytrium* sp. batch cultivation running through growth, transition and lipid accumulation phases (Figure 7). Thraustochytrids and other oleaginous model systems are analytically challenging due to the large changes in the biomass composition during the cultivation phases, and the culture potentially contains molecules that interfere with the downstream analytical steps. The mass spectrometer is a concentration dependent detector and highly affected by interfering substances [39,40]. Thus, all sampling, extraction and sampling processing protocols needed adjustments and validation for the *Aurantiochytrium* samples. This included optimization of sample size and several sample processing steps including removing potential interfering compounds, e.g., high amount of lipids. The use of ^13^C internal standard dilution strategy is necessary to provide the quantitative data at the highest level of accuracy and precision [35]. We observed many instances of interfering coeluting compounds that reduce analytical quality if not compensated for by the ^13^C or non-natural ^12^C internal standards. However, even for this high lipid containing model system we were able to obtain satisfactory analytical precision at the same level as other model systems (*Escherichia coli*, yeast and human cell lines) for most methods and analytes. The main exception was the poor chromatographic resolution of the hexose-phosphates. The capillary ion chromatograph provides baseline separation of most hexose-phosphate [41,42] for all other tested model systems, but not for thraustochytrid samples. The reasons are unknown and even careful removing of hydrophobic entities by solid phase extraction purification and 3 KDa spin filtering did not result in hexose-phosphate base line separation. The NAD and CoA metabolites are very challenging analytes and we included a separate paragraph in Section 4 presenting our experience and needed adaptation of the LC-MS/MS methods for thraustochytrid extracts. 

The large decrease over time in the intracellular concentrations for most metabolites introduces biases when using multivariate data analysis, ratio comparisons, etc. The chosen unit (μmol/g DW) is the most obvious one to merge data from different analytical methods for this model system as cell sizes and intracellular volume were not possible to measure due to strong cell clustering/pelleting throughout the cultivation. We tried various ways of normalization (both to sum of metabolite concentrations and to the lipid-free intracellular volume) and since all attempts returned the same trends, we can be confident that the results presented in the PCA score plot (Figure 4B) and the log_2_ ratio plot (Figure 6) represent, at least qualitatively, the true changes in *Aurantiochytrium* sp. from growth and transition into the strict LA phase. 

The quantitative aspect of the SFC lipid analysis is also challenging since the extract is so largely dominated by TGs. The highest precision would be obtained by analyzing the same sample with two dilutions, a high for TG and a low for the other lipid classes. However, that will reduce throughput and still maintain the challenge with quantitative merging of the data [33]. In any case, the applied lipid profiling method is only semiquantitative and returned interesting results on individual lipid levels for a relative evaluation and interpretation. Interestingly, the data showed that DHA can be incorporated into all three acyl side chains in TG. Thus, DHA production and LA can still proceed if FAS activity is selectively inhibited since it will not be strictly needed after cell division and lipid membrane synthesis cease (confer Figure 1 for biosynthetic pathways). Thus, a DHA-enriched product can be obtained and ideally at the same total LA rates since all cellular resources are available for DHA production, but this will be a challenging strain engineering task. The cells need to direct their resources to different processes in the growth and in the LA phase. The trigger for LA in the present cultivation condition is N-depletion. Interestingly, N-depletion is not that dramatic on the total respiratory rate (as observed on the off-gas CO_2_ profile) as usually is observed for non-oleaginous microorganisms. The usual response is a 70–80% immediate decrease in CO_2_ production, and leveling off at this low level for the rest of the cultivation, e.g., for *Streptomyces* [43]. In cultivation of strain T66, the CO_2_ concentration in the off gas peaked around 5 h after N-depletion, followed by a slow decrease, in accordance with the growth profile when cultivated on glycerol and glutamate as C- and N-sources [16,43]. One reason may be that thraustochytrids have a relatively low growth rate (μ_max_ around 0.2 h^−1^ in mineral medium) [16].

There are some similar studies on oleagineous model systems including thraustochytrids [29,30,31,32,44,45] but none covering with quantitative methods the whole central carbon metabolism, redox-cofactors and lipid composition as completely as presented here. Those studies have mostly applied untargeted LC-MS and GC-MS profiling, few internal standards and usually only normalized to the total peak area. Geng and coworkers used qualitative GC-MS analysis and reported 4-aminobutyric acid, proline and glutamine as potential biomarkers associated with growth and lipid accumulation in an engineered *Schizochytrium* strain [29]. Perez and coworkers used both LC-MS and GC-MS operated in nontarget qualitative mode to profile the metabolome of acetate and glucose grown *Aurantiochytrium limacinum* SR21 cells, and especially TCA metabolites were upregulated on acetate [30]. Pei and coworkers combined transcriptome and qualitative GC-MS analysis in a *Crypthecodinium* sp. and reported upregulated metabolites both in glycolysis and TCA but low correlation to the transcriptome [45]. Yang and coworkers studied *Schizochytrium* strains with different DHA content and reported downregulated glycolytic and upregulated TCA pools in the high DHA strain [31]. Liu and coworkers applied GC-MS and LC-MS metabolite profiling to maintain high DHA productivity during repeated fed-batch cultivation of *Crypthecodinium* [32]. All these studies provide interesting information on the relative cellular adjustments to changes in cultivation conditions and growth stages which can be used to further improve yields and productivities of the DHA-bioprocess. However, the studies are difficult to compare, also with ours, since strains and cultivation conditions vary, and, as important, large differences in analytical approach. However, all these studies present far from the complete picture of the central metabolism; thus, multiple MS methods with individual internal standards have to be applied for a more comprehensive and quantitative coverage of the metabolome [34,46]. This is needed for advancing the knowledge of how the central metabolism maintains the supply of carbon precursors and reducing cofactors during growth as well as the lipid accumulation phase. Our observation of dramatically decreasing metabolite pools is also important for the data evaluation and interpretation strategies.

The data presented here provides novel insight and identifies more likely metabolite bottlenecks in the fatty acid synthesis and lipid accumulation (Figure 1). When the cell growth ceases, more intracellular resources, like acetyl-CoA and NADPH, become available for LA, as verified by the MS results showing increased pools of these. Thus, the metabolite profiling data points more to the decreased pools of LC-CoAs and the lipid head group glycerol 3-P as limiting precursors and cause for the drop in the lipid synthesis rate in the LA phase (25–45 h Figure 2) vs. the growth phase (see log_2_ ratios between the LA and growth phases in Figure 6). Palmitic acid (C16:0) and DHA are the two dominating fatty acids in the lipid pool, 30% and 20%, respectively, of the fatty acids in the total lipid extract (Figure 3B). It is interesting to note that the PKS generated DHA-CoA is present at a 10 times higher concentration than the FAS generated C16:0-CoA (Figure 5B), this leaves room for speculation of acyl chain CoA affinities and preferences of the multiple mono-/di-acylglycerol acyltransferases in the *Aurantiochytrium* genome [6,12] that catalyze the two last steps in the triacylglycerol synthesis pathway. Heggeset et al. reported that FAS genes were significantly upregulated and PKS synthase moderately upregulated under N-deficient conditions [6]; but only analysis at the proteome level can enlighten if the decline in synthesis is due to less available PKS/FAS-enzymes, precursors or reducing cofactors. Even though the nucleotide phosphate and amino acid pools were downregulated (Figure 4A, Supplementary Figure S1), all metabolite species were detected; thus, both gene transcription and translation could in principle proceed for maintaining the balance between enzyme degradation and new synthesis. Further experimentation at multi-omics levels and at multiple cultivation conditions are needed to enlighten this.

## 4. Materials and Methods

Figure 7 presents the cultivation, sampling and analytical approach established and applied for this high DHA producing model system.

### 4.1. Cultivations

*Auranthiochytrium* sp. T66 was cultivated in 3 L Eppendorf BioFlo 115 bioreactors with an operating volume of 1.5 L. The medium consisted of (in g/L) glucose (50), KH_2_PO_4_ (2.0), NH_4_Cl (2.0), Na_2_SO_4_ (18), MgSO_4_ × 7H_2_O (1.2), KCl (0.4), CaCl_2_ × 2H_2_O (0.5), and yeast extract (0.1) dissolved in water (ion free). The medium was supplemented with 1.125 mL of vitamin solution (in g/L: 0.005 biotin, 0.1 folic acid, 0.1 Ca-panthotenat, 0.1 thiamin HCl, and 0.005 B12 cobalamin) and 0.75 mL of trace mineral solution (in mg/L: 5000 FeSO_4_ × 7H_2_O, 440 ZnSO_4_ × 7H_2_O, 390 CuSO_4_ × 5H_2_O, 150 MnSO_4_ × 7H_2_O, 10 NaMoO_4_ × 2H_2_O, 20 CoCl_2_ × 6H_2_O). Medium components were purchased from Sigma-Aldrich, St. Louis, MO, USA.

The cultivation was operated at 30 °C, pH 7.0 and with an air flow rate of 0.5 VVM. Offgas composition was monitored by a DasGip GA4 exhaust level analyzer. Level of dissolved oxygen was controlled with agitation cascade and was never below 20% of saturation. Inoculum (3% *v*/*v*) for bioreactor cultivations was prepared in a two-step shake flask procedure, first step grown overnight and second step grown until OD 6 ensuring that the inoculum culture was in active growth phase when transferred. First step inoculum medium was composed of (in g/L) 30 glucose, 0.3 KH_2_PO_4_, 8.0 Na-glutamate, 5.0 yeast extract, 18 Na_2_SO_4_, 0.25 MgSO_4_ × 7H_2_O, 0.4 KCl, 6.1 Tris-base, 5.8 maleic acid, and second step (in g/L): 30 glucose, 0.3 KH_2_PO_4_, 2.0 NH4Cl, 18 Na_2_SO_4_, 0.25 MgSO_4_ × 7H_2_O, 0.4 KCl, 6.1 Tris-base, 5.8 maleic acid, 0.1 mL vitamin solution, and 0.1 mL trace mineral solution (same composition as for bioreactor cultivations). The shake flasks were incubated at 30 °C and 200 rpm.

### 4.2. Biomass and Extracellular Analyses

Sampling volumes varied throughout the cultivation and were optimized to the individual downstream analysis. Samples for dry weight (either 2, 5, 10, or 20 mL) were centrifuged twice and washed with 1.5% NaCl solution and dried at 120 °C overnight. Glucose concentration and potential organic acids produced and excreted into the supernatant during the cultivation were analyzed on a Waters Alliance HPLC system equipped with UV and RI detector. An Agilent Hi-Plex H 7.7 × 300 mm, 8 mm HPLC column was used with 0.05 M H_2_SO_4_ mobile phase at 0.6 mL/min flow rate. 

### 4.3. Fatty Acid and Lipid Profiling

#### 4.3.1. Lipid Extraction

A Precellys^®^24 bead homogenizer with a Cryolys temperature controller (all Bertin Technologies SAS, Montigny-le-Bretonneux, France), was employed for homogenization and a solvent system previously described by Blight and Dyer was applied for lipid extraction [47]. Twenty milligrams of dried pellet were accurately weighed and homogenized with zirconium oxide beads (0.5 ± 0.01 g, Ø 1.4 mm) in 1.0 mL of a cold mixture of chloroform:methanol (1:2, *v*/*v*). Three bead-beating cycles at 6000 rpm for 30 s with 15 s intermediate pause were applied to obtain a homogenous sample. An addition of 0.333 mL of a cold chloroform followed and tubes were vortexed for 20 s. Phase separation was induced by adding 0.333 mL of water. A phase separation was accelerated by a short and mild centrifugation cycle. A chloroform layer containing lipids was collected and cleared of cell debris with a syringe filter with PTFE membrane, 0.2 um, Ø 13 mm (VWR International). Final extracts were stored at −30 °C in glass vials until analyzed with ultra-performance convergence chromatography with high-resolution mass spectrometry (UPC2-ESI-QTOF). Dichloromethane was used as a sample diluent prior to injection. An aliquot of the lipid extract was transferred to a preweighted glass vial and evaporated on a heating block 40 °C while flushing with N_2_ gas for ca. 20 min to determine the amount of total lipids.

#### 4.3.2. Nontarget Lipid Analysis

Detailed information about the lipid profiling method can be found in the recent publication by Bartosova and coworkers [33]. A lipid profile analysis was performed using an UPC^2®^ separation system coupled to a hybrid quadrupole orthogonal time-of-flight mass spectrometer SYNAPT G2-S HDMS (both Waters, Milford, MA, USA). The UPC^2®^ system was equipped with a binary pump, a convergence manager, a column heater, an autosampler, and an auxiliary pump. The separation system was coupled to the MS via a flow splitter kit that consisted of two T-pieces allowing control of the backpressure and infusion of a make-up liquid. The make-up liquid consisted of methanol:isopropanol:water (50:49:1, *v*/*v*/*v*) and its flow rate was set to 0.2 mL/min. Pressurized CO_2_ was used as mobile phase A, methanol:water (99:1, *v*/*v*) with 30 mM ammonium acetate was used as mobile phase B. The gradient of mobile phase B followed the scheme: 0 min, 1%; 4.0 min, 30% (6); 4.4 min, 50% (2); 6.25 min 50% (1); 7.25 min, 50% (6); 7.35 min, 1% (6), 8.50 min, 1%. The column temperature was 50 °C, flow rate 1.9 mL/min and automated back-pressure regulator (ABPR) was set to 1800 psi. The mass spectrometer was equipped with an ESI source operated in positive mode. A data independent acquisition technique, MS^E^, was applied for data acquisition and the collision energy ramped from 20 to 30 eV. The MS tuning parameters were set as follows: capillary voltages 3.0 kV, the source temperature 150 °C, the sampling cone 40 V, the source offset 60 V, the desolvation temperature 500 °C, the cone gas flow 50 L/h, the desolvation gas flow 850 L/h, and the nebulizer gas pressure 4 bar. Data were acquired over the mass range of 50–1200 Da and resolution of mass spectrometer was 20,000. The lipid data was normalized to the total lipid content in the biomass.

#### 4.3.3. Hydrolysis of Lipids

An aliquot of lipid extract (0.3 mL) was transferred to a glass tube and lipids were hydrolyzed with 1 mL of 2 M KOH (prepared in 80% ethanol). Tubes were flushed with nitrogen and incubated at 70 °C for 90 min. K-salts were precipitated by addition of 1 mL of 4 M H_2_SO_4_. Fatty acids were extracted with 2 mL of dichloromethane. Tubes were vortexed for 60 s and then centrifuged at 800× *g* for 5 min. An aliquot of the lower organic phase was transferred into dark glass HPLC vials. Vials with samples were flushed with nitrogen, capped with PTFE lined caps and stored at −30 °C until analyzed with ultra-performance convergence chromatography tandem mass spectrometry (UPC^2^-MS/MS).

#### 4.3.4. Quantitative Analysis of Fatty Acids

A quantitative analysis of fatty acids was carried out using an UPC^2®^ separation system coupled to a tandem quadrupole mass spectrometer Xevo TQ-S (both Waters, Milford, MA, USA). The separation was performed using an Acquity UPC2 Torus 1-AA (1.7 mm, 3.0 × 100 mm) column, the column temperature was 50 °C. CO_2_ was used as a mobile phase A, methanol containing 0.1% HCOOH was used as a mobile phase B and methanol containing 0.1% NH_4_OH was used as a make-up liquid. The gradient of the mobile phase B was set as follows: 0 min, 2%; 5.0 min, 8% (9); 5.5 min, 30% (1); 6.5 min 30% (11); 8.0 min, 2% (1). The flow rate of mobile phase and make-up liquid was 1.5 and 0.22 mL/min, respectively. The back-pressure regulator was set to 1700 psi. Negative ion electrospray ionization mode was applied and the MS tuning parameters were set as follows: capillary voltage −2.0 kV, the source temperature 150 °C, the sampling cone 30 V, the desolvation temperature 550 °C, the cone gas flow 150 L/h, the desolvation gas flow 1000 L/h, and the nebulizer gas pressure 4 bar. The mass spectrometer was operated in MRM mode (details in Supplementary Table S2).

Individual stock solutions of free fatty acids were prepared in methanol. A series of standard mixes in the range of 15–3000 nM, prepared in dichloromethan, were used to build up calibration curves, each standard solution contained tridecanoic acid, ^13^C_16_-palmitic acid and ^13^C_18_-oleic acid as ISTDs (100 nM each). ISTD mix was added into the samples prior to analysis.

### 4.4. Metabolite Profiling

Some of the metabolite profiling methods presented in Figure 7B have been published earlier but two new were established as part of this work (SC-CoA and LC-CoA). MS transition information for all methods were collected and presented in Supplementary Table S5. All methods employ ^13^C internal standard for individual metabolites (either using *E. coli* or yeast extracts or from commercially available and complete amino acid and organic acid mixtures). This improves analytical accuracy and precision which, in addition to ease data interpretation and evaluation, is also necessary during validation of sampling, extraction and stability of samples (see [42,48] for more detailed presentation and evaluation of these aspects). Multivariate analysis was performed with MetaboAnalyst online software [49].

Intracellular concentration of amino acids, organic acids, sugar phosphates and other phosphorylated metabolites, and nucleotides were determined with the two LC-MS/MS and one capillary ion chromatography-MS/MS method as described earlier [34,41,42]. Oxidized and reduced pyridine metabolites (NADs) were quantified with the recently established zwitterionic HILIC-MS/MS method [48]. Sample volumes needed optimization for the thraustochytrid biomass to fit the linear range of the calibration curves, and the following sampling volumes were used for the intracellular metabolite analysis: 10 mL for sampling points 1 and 2, 5 mL for 3 and 4, 2 mL for 5 and 6, irrespective of the subsequent analytical method.

#### 4.4.1. Extraction and Quantitation of CoA-Metabolites

Quantitative LC-MS/MS methods for short chain (SC) CoAs and long chain (LC) CoAs have not published earlier and detailed information is provided below. 

Acetyl-CoA, CoA, Malonyl-CoA and Succinyl-CoA were extracted in two-steps using a mixture of acetonitrile:methanol:water (70:10:20, *v*/*v*/*v*) containing 15 mM ammonium acetate pH 5.0. The pellets were thawed on ice and thoroughly suspended in 1.5 mL of cold extraction mixture. An aliquot of the pellet suspension (0.4 mL) was transferred to a clean Eppendorf tube and extraction was performed by constant shaking for 5 min (1 °C, 1500 rpm) using a thermo-shaker (Thermal shake lite, VWR, Radnor, PA, USA). The extracts were cleared of cell debris by centrifugation at 4500× *g* for 2 min at 4 °C. The supernatant was filtered using 3 kDa molecular weight cut-off filters (516–0228, VWR) and centrifuged at 14,000× *g* for 5 min at 4 °C, while the pellet residual was resuspended with 0.4 mL of the extraction mixture and underwent a second extraction step by shaking at 1500 rpm for 5 min at 1 °C. The second extract was cleared of debris in two consecutive centrifugation steps as described above and the final extracts were pooled. Ten percent of ^13^C labeled ISTD mixture of CoAs was added to an aliquot of the final sample extract and samples were immediately analyzed using UPLC-TQ-XS. ^13^C labeled ISTD mixture of CoAs was extracted from *E. coli* cultured with ^13^C_6_-glucose (>99%, 389374, Merck, Darmstadt, Germany) the same way as described above, but the volume of the extraction solvent needs to be optimized with respect to the actual content of SC-CoAs in the starting material used. Preparation of the *E. coli* culture and pelleting procedure is described in detail by L. M. Røst et al. [34].

Analysis of the SC-CoAs was performed using an UPLC I-Class separation system coupled to a triple quadrupole mass spectrometer Xevo TQ-XS (both Waters, Milford, MA, USA). Separation was conducted on a SeQuant^®^ ZIC-HILIC (3 μm, 100 × 2.1 mm) column, the column temperature was 40 °C. Mobile phase A consisted of acetonitrile:water (50:50, *v*/*v*) and mobile phase B consisted of acetonitrile:water (75:25, *v*/*v*), both contained 50 mM CH_3_COONH_4_ pH 5. The gradient of mobile phase B was set as follows: 0 min, 99.9%; 1.0 min, 30% (6); 4.4 min, 99.9% (6); 6.25 min 50% (6); 1.5 min, 35% (6); 5.0 min, 0.1% (6), 6.0 min, 0.1% (6), 6.1 min, 99.9% (6), 8.0 min, 99.9% (6). The flow rate of the mobile phase was 0.4 mL/min. Positive ion electrospray ionization mode was applied and the MS tuning parameters were set as follows: capillary voltage 1.5 kV, the source temperature 150 °C, the sampling cone 45 V, the desolvation temperature 500 °C, the cone gas flow 150 L/h, the desolvation gas flow 1000 L/h, and the nebulizer gas flow 5 bar. The mass spectrometer operated in MRM mode and relevant parameters are presented in Supplementary Table S5.

Individual solutions of SC-CoA-metabolites were prepared in water. A series of standard mixes in the range of 100–8000 nM, prepared in the extraction solvent, were used to build up calibration curves, each standard solution contained 10% ^13^C-labeled *E. coli* extract. The stability of SC-CoA-metabolites, particularly succinyl-CoA, is limited, thus all extracts and standards need to be prepared freshly on the day of analysis. 

The extraction procedure for long chain (LC) CoA-metabolites was adapted from Minkler P. E. et al. [50] and is based on liquid extraction followed by isolation with anion-exchange SPE using columns packed with 100 mg 2-(2-pyridyl)ethyl functionalized silica gel Supelco, (Bellefonte, PA, USA). After collection, the cell pellets were centrifuged and washed once with solution of NaCl (15 g/L) and then stored at −80 °C until extraction and analysis. Pellets were thawed on ice and centrifuged briefly at 4 °C to remove surplus of washing buffer. The pellets were then thoroughly suspended with 2.2 mL of acetonitrile/isopropanol (3:1, *v*/*v*) containing 250 nM of an internal standard 17:0-CoA. An aliquot of 0.9 mL was transferred to a tube containing 0.75 g of zirconium oxide beads (Ø 1.4 mm) and homogenized for 2 × 20 s at 6500 rpm with a Precellys homogenizer equipped with a Cryolys cooling system (both Bertin, Montigny-le-Bretonneux, France). Then 0.3 mL of 0.1 M KH_2_PO_4_ (pH = 6.7) was added into the tube and after a short vortex step the tubes were centrifuged for 10 min at 14,000 rpm and 4 °C. An aliquot of 0.8 mL was transferred to a tube containing 0.2 mL of glacial acetic acid.

The SPE cartridge was conditioned with 1 mL of acetonitrile/isopropanol/water/acetic acid (9:3:4:4, *v*/*v*/*v*/*v*) prior to application of the acidified supernatant (1 mL). The SPE column was washed with 1 mL of acetonitrile/isopropanol/water/acetic acid (9:3:4:4) to remove unretained species. LC-CoAs were eluted with 2 × 0.5 mL of methanol/250 mM ammonium formate (pH = 7.0), (4:1, *v*/*v*). The extracts were analyzed directly.

Analysis of LC-CoAs was performed using an UPLC I-Class separation system coupled to a tandem quadrupole mass spectrometer Xevo TQ-S (both Waters, Milford, MA, USA). Separation was conducted on an ACQUITY BEH C18 (1.7 μm, 100 × 2.1 mm) column and temperature was kept at 25 °C. Mobile phase A consisted of water containing 0.025% NH_4_OH and mobile phase B consisted of acetonitrile containing 0.025% NH_4_OH. The gradient of mobile phase B was set as follows: 0 min, 20%; 5.0 min, 50% (6); 5.5 min, 85% (6); 7.0 min 85% (6); 7.5 min, 20% (6); 9.5 min, 20%. The flow rate of the mobile phase was 0.22 mL/min and injection volume was 2 uL. Positive ion electrospray ionization mode was applied and the MS tuning parameters were set as follows: capillary voltage 3.0 kV, the source temperature 150 °C, the sampling cone 50 V, the desolvation temperature 350 °C, the cone gas flow 150 L/h, the desolvation gas flow 900 L/h, and the nebulizer gas flow 6 bar. The mass spectrometer operated in MRM mode and relevant parameters are presented in Supplementary Table S5.

Individual stock solutions of LC-CoA-metabolites were prepared in a mixture of methanol:water (4:1, *v*/*v*). A series of standard mixes in the range of 25–400 nM (25–2500 nM for DHA-CoA), prepared by standard addition to biological extracts containing 17:0-CoA as an internal standard, were used to build up calibration curves. The stability of LC-CoA-metabolites, particularly of polyunsaturated fatty acyl-CoAs, is limited, thus it is advisable to prepare all standards and extracts freshly on the day of analysis, however, if necessary, it is possible to store standards and extracts overnight at −80 °C. Samples placed in an autosampler at 6 °C for more than 7 h showed a loss of response corresponding to approximately 10%.

#### 4.4.2. Comments on the Optimization of Extraction and Quantitation of the NAD- and SC-CoA-Metabolites

Thraustochytrids produce high amounts of biomass rich in lipids, which might be particularly demanding when quantitative extraction of intracellular metabolites is of interest. Moreover, both NAD- and CoA-metabolites represent challenges in terms of their stability. The extraction procedure for NAD-metabolites, originally optimized for *E. coli* and human cells, was adapted from [48]. The method utilizes isotope dilution to compensate matrix effects and potential degradation of analytes, ^13^C-labeled isotopologues of NAD-metabolites were obtained from *E. coli* cultured with ^13^C_6_-glucose. We also applied the same isotope dilution strategy analysis in this study; but the extraction procedure of NAD-metabolites from thraustochytrids needed additional optimization in terms of the extraction time, the extraction solvent volume, matrix effects, and potential interferences coming from the high lipid content (data not shown). First of all, we had to increase the volume of extraction solvent to ensure proper resuspension of the cell pellet and obtain homogenous and representative samples. It was found the pellets need to be resuspended with at least 0.6 mL of a cold extraction solvent. For extraction, an aliquot of 0.270 mL was subsequently transferred to a clean tube containing 0.030 mL of ^13^C-labeled ISTD extract of *E. coli*. It was found the extraction time 5 min was sufficient, neither an extended extraction time nor repeated extraction steps improved the final yield. NAD-metabolites were stable for 24 h in the autosampler kept at 6 °C, the response of all NAD-metabolites was effectively compensated by isotope dilution method. In addition, we prepared two calibration series to investigate matrix effects on the response of analytes. The slopes of the calibration curves prepared in extraction solvent were compared to the slopes of calibration curves prepared by standard addition to whole cell extract of thraustochytrids and we obtained different slopes for NADP, NADPH and FAD. Thus, we recommend using matrix matched calibration for the metabolites mentioned.

The extraction procedure and composition of the extraction solvent for CoA-metabolites also needed optimization. The pH was slightly acidic to favor stability of the target analytes and contained higher amount of ammonium acetate buffer to match the initial chromatographic condition. The extraction procedure was optimized in terms of extraction time, extraction solvent volume and matrix effects (data not shown). Similar to the extraction of NADs, 5 min were sufficient for extraction and extended extraction time did not increase the yields, but a repeated extraction step was inevitable to obtain complete extract. A third extraction step was not necessary since the response after the third extraction cycle corresponded to approx. 10% of the original signal obtained after the first extraction step. At least 1.2 mL of a cold extraction solvent is needed for pellet suspension, the volume of 0.6 mL or lower led to nonreproducible results. Here, we also utilized the isotope dilution method as described above to compensate potential matrix effects and degradation of CoA-metabolites, however, the ^13^C-labeled ISTD extract was added into the samples right after the extraction. CoA and acetyl-CoA were stable in the autosampler maintained at 6 °C for at least 24 h, while succinyl-CoA is more susceptible to degradation; thus, its analysis should be performed within 8 h after extraction. Potential matrix effect on response was also explored and similarity of slopes of calibration series prepared in extraction solvent and in the whole cell extract were compared. All the slopes were in agreement and therefore calibration curves prepared in extraction solvent can be used for accurate quantification of SC-CoA-metabolites.

## 5. Conclusions

A high-resolution phenotyping at metabolite and lipid levels to generate deeper insight into the thraustochytrid cell factory with particular focus on central carbon, lipid biosynthesis pathway and redox metabolism was presented. The data indicates that fatty acid synthesis is not limited by the acetyl-CoA precursor and NADPH availability but rather points to decline in lipid head group glycerol-3-phosphate and long chain-CoAs pools as potential bottlenecks and causes for the gradual lowering of the LA rates.

## Data Availability

All processed data is available in Supplementary materials. Information about design of quantitative analysis including external standard series and ^13^C internal standards and how raw data files are processed can be obtained by contacting corresponding author.

## Supplementary Materials

The following are available online at https://www.mdpi.com/2218-1989/11/3/135/s1, Figure S1: Relative intracellular metabolite concentrations, Table S1: Cultivation data, Table S2: Fatty acid content in lipid extract, Table S3: Top 10 lipid species, Table S4: Absolute metabolite concentrations, Table S5: MRM settings for the metabolite profiling methods.

## References

[1] Aasen I.M., Ertesvåg H., Heggeset T.M.B., Liu B., Brautaset T., Vadstein O., Ellingsen T.E. (2016). Thraustochytrids as production organisms for docosahexaenoic acid (DHA), squalene, and carotenoids. Appl. Microbiol. Biotechnol..

[2] Marchan L.F., Chang K.J.L., Nichols P.D., Mitchell W.J., Polglase J.L., Gutierrez T. (2018). Taxonomy, ecology and biotechnological applications of thraustochytrids: A review. Biotechnol. Adv..

[3] Colonia B.S.O., Pereira G.V.D.M., Soccol C.R. (2020). Omega-3 microbial oils from marine thraustochytrids as a sustainable and technological solution: A review and patent landscape. Trends Food Sci. Technol..

[4] Patel A., Karageorgou D., Rova E., Katapodis P., Rova U., Christakopoulos P., Matsakas L. (2020). An Overview of Potential Oleaginous Microorganisms and Their Role in Biodiesel and Omega-3 Fatty Acid-Based Industries. Microorganisms.

[5] Morabito C., Bournaud C., Maës C., Schuler M., Cigliano R.A., Dellero Y., Maréchal E., Amato A., Rébeillé F. (2019). The lipid metabolism in thraustochytrids. Prog. Lipid Res..

[6] Heggeset T.M.B., Ertesvåg H., Liu B., Ellingsen T.E., Vadstein O., Aasen I.M. (2019). Lipid and DHA-production in *Aurantiochytrium* sp.—Responses to nitrogen starvation and oxygen limitation revealed by analyses of production kinetics and global transcriptomes. Sci. Rep..

[7] Iwasaka H., Koyanagi R., Satoh R., Watanabe K., Hisata K., Satoh N., Aki T. (2018). A Possible Trifunctional beta-Carotene Synthase Gene Identified in the Draft Genome of *Aurantiochytrium* sp. Strain KH105. Genes.

[8] Dellero Y., Cagnac O., Rose S., Seddiki K., Cussac M., Morabito C., Lupette J., Cigliano R.A., Sanseverino W., Kuntz M. (2018). Proposal of a new thraustochytrid genus Hondaea gen. nov and comparison of its lipid dynamics with the closely related pseudo-cryptic genus *Aurantiochytrium*. Algal Res. Biomass Biofuels Bioprod..

[9] Chen W., Zhou P.-P., Zhang M., Zhu Y.-M., Wang X.-P., Luo X.-A., Bao Z.-D., Yu L.-J. (2016). Transcriptome analysis reveals that up-regulation of the fatty acid synthase gene promotes the accumulation of docosahexaenoic acid in *Schizochytrium* sp S056 when glycerol is used. Algal Res. Biomass Biofuels Bioprod..

[10] Yue X.-H., Chen W.-C., Wang Z.-M., Liu P.-Y., Li X.-Y., Lin C.-B., Lu S.-H., Huang F.-H., Wan X. (2019). Lipid Distribution Pattern and Transcriptomic Insights Revealed the Potential Mechanism of Docosahexaenoic Acid Traffics in *Schizochytrium* sp. A-2. J. Agric. Food Chem..

[11] Hauvermale A., Kuner J., Rosenzweig B., Guerra D., Diltz S., Metz J.G. (2006). Fatty acid production in *Schizochytrium* sp.: Involvement of a polyunsaturated fatty acid synthase and a type I fatty acid synthase. Lipids.

[12] Liu B., Ertesvåg H., Aasen I.M., Vadstein O., Brautaset T., Heggeset T.M.B. (2016). Draft genome sequence of the docosahexaenoic acid producing thraustochytrid *Aurantiochytrium* sp. T66. Genom. Data.

[13] Zhao X.M., Qiu X. (2018). Analysis of the biosynthetic process of fatty acids in Thraustochytrium. Biochimie.

[14] Ratledge C. (2014). The role of malic enzyme as the provider of NADPH in oleaginous microorganisms: A reappraisal and unsolved problems. Biotechnol. Lett..

[15] Chen C.Y., Yang Y.T. (2018). Combining engineering strategies and fermentation technology to enhance docosahexaenoic acid (DHA) production from an indigenous *Thraustochytrium* sp BM2 strain. Biochem. Eng. J..

[16] Jakobsen A.N., Aasen I.M., Josefsen K.D., Strøm A.R. (2008). Accumulation of docosahexaenoic acid-rich lipid in thraustochytrid *Aurantiochytrium* sp strain T66: Effects of N and P starvation and O (2) limitation. Appl. Microbiol. Biotechnol..

[17] Guo D.-S., Ji X.-J., Ren L.-J., Li G.-L., Sun X.-M., Chen K.-Q., Gao S., Huang H. (2018). Development of a scale-up strategy for fermentative production of docosahexaenoic acid by *Schizochytrium* sp.. Chem. Eng. Sci..

[18] Guo D.S., XJ J., LJ R., GL L., FW Y. (2016). Development of a real-time bioprocess monitoring method for docosahexaenoic acid production by *Schizochytrium* sp.. Bioresour. Technol..

[19] Janthanomsuk P., Verduyn C., Chauvatcharin S. (2015). Improved docosahexaenoic acid production in *Aurantiochytrium* by glucose limited pH-auxostat fed-batch cultivation. Bioresour. Technol..

[20] Kim K., Kim E.J., Ryu B.-G., Park S., Choi Y.-E., Yang J.-W. (2013). A novel fed-batch process based on the biology of *Aurantiochytrium* sp KRS101 for the production of biodiesel and docosahexaenoic acid. Bioresour. Technol..

[21] Wang Q.Z., Ye H., Sen B., Xie Y., He Y., Park S., Wnag G. (2018). Improved production of docosahexaenoic acid in batch fermentation by newly-isolated strains of *Schizochytrium* sp. and Thraustochytriidae sp. through bioprocess optimization. Synth. Syst. Biotechnol..

[22] Xu X., Huang C., Xu Z., Xu H., Wang Z., Yu X. (2020). The strategies to reduce cost and improve productivity in DHA production by *Aurantiochytrium* sp.: From biochemical to genetic respects. Appl. Microbiol. Biotechnol..

[23] Sun X.-M., Ren L.-J., Zhao Q.-Y., Ji X.-J., Huang H. (2019). Enhancement of lipid accumulation in microalgae by metabolic engineering. Biochimica Et Biophysica Acta-Mol. Cell Biol. Lipids.

[24] Cui G.-Z., Ma Z., Liu Y.-J., Feng Y., Sun Z., Cheng Y., Song X., Cui Q. (2016). Overexpression of glucose-6-phosphate dehydrogenase enhanced the polyunsaturated fatty acid composition of *Aurantiochytrium* sp SD116. Algal Res. Biomass Biofuels Bioprod..

[25] Sakaguchi K., Matsuda T., Kobayashi T., Ohara J.-I., Hamaguchi R., Abe E., Nagano N., Hayashi M., Ueda M., Honda D. (2012). Versatile transformation system that is applicable to both multiple transgene expression and gene targeting for Thraustochytrids. Appl. Env. Microbiol..

[26] Merkx-Jacques A., Rasmussen H., Muise D.M., Benjamin J.J.R., Kottwitz H., Tanner K., Milway M.T., Purdue L.M., Scaife M.A., Armenta R.E. (2018). Engineering xylose metabolism in thraustochytrid T18. Biotechnol. Biofuels.

[27] Faktorová D., Nisbet R.E.R., Robledo J.A.F., Casacuberta E., Sudek L., Allen A.E., Ares M., Aresté C., Balestreri C., Barbrook A.C. (2020). Genetic tool development in marine protists: Emerging model organisms for experimental cell biology. bioRxiv.

[28] Sun H., Chen H., Zang X., Hou P., Zhou B., Liu Y., Wu F., Cao X., Zhang X. (2015). Application of the Cre/loxP Site-Specific Recombination System for Gene Transformation in *Aurantiochytrium limacinum*. Molecules.

[29] Geng L., Chen S., Sun X., Hu X., Ji X., Huang H., Ren L. (2019). Fermentation performance and metabolomic analysis of an engineered high-yield PUFA-producing strain of *Schizochytrium* sp.. Bioprocess. Biosyst. Eng..

[30] Perez C.M.T., Watanabe K., Okamura Y., Nakashimada Y., Aki T. (2019). Metabolite Profile Analysis of *Aurantiochytrium limacinum* SR21 Grown on Acetate-based Medium for Lipid Fermentation. J. Oleo. Sci..

[31] Yang J., Song X., Wang L., Cui Q. (2020). Comprehensive Analysis of Metabolic Alterations in *Schizochytrium* sp. Strains with Different DHA Content. J. Chromatogr. B.

[32] Liu L., Wang F., Pei G., Cui J., Diao J., Lv M., Chen L., Zhang W. (2020). Repeated fed-batch strategy and metabolomic analysis to achieve high docosahexaenoic acid productivity in *Crypthecodinium cohnii*. Microb. Cell Factories.

[33] Bartosova Z., Gonzalez S.V., Voigt A., Bruheim P. (2020). High. throughput semi-quantitative UHPSFC-MS/MS lipid profiling and lipid class determination. J. Chromatogr. Sci..

[34] Røst L.M., Thorfinnsdottir L.B., Kumar K., Fuchino K., Langørgen I.E., Bartosova Z., Kristiansen K.A., Bruheim P. (2020). Absolute Quantification of the Central Carbon Metabolome in Eight Commonly Applied Prokaryotic and Eukaryotic Model Systems. Metabolites.

[35] Seifar R.M., Ras C., van Dam J.C., van Gulik J.C., Heijnen J.J., van Winden W.A. (2009). Simultaneous quantification of free nucleotides in complex biological samples using ion pair reversed phase liquid chromatography isotope dilution tandem mass spectrometry. Anal. Biochem..

[36] Chubukov V., Gerosa L., Kochanowski K., Sauer U. (2014). Coordination of microbial metabolism. Nat. Rev. Microbiol..

[37] Huergo L.F., Dixon R. (2015). The Emergence of 2-Oxoglutarate as a Master Regulator Metabolite. Microbiol. Mol. Biol. Rev..

[38] Droste P., Miebach S., Niedenführ S., Wiechert W., Nöh K. (2011). Visualizing multi-omics data in metabolic networks with the software Omix-A case study. Biosystems.

[39] Bruins A.P. (1998). Mechanistic aspects of electrospray ionization. J. Chromatogr. A.

[40] Zhou W.L., Yang S., Wang P.G. (2017). Matrix effects and application of matrix effect factor. Bioanalysis.

[41] Kvitvang H.F.N., Kristiansen K.A., Bruheim P. (2014). Assessment of capillary anion exchange ion chromatography tandem mass spectrometry for the quantitative profiling of the phosphometabolome and organic acids in biological extracts. J. Chromatogr. A.

[42] Stafsnes M.H., Røst L.M., Bruheim P. (2018). Improved phosphometabolome profiling applying isotope dilution strategy and capillary ion chromatography-tandem mass spectrometry. J. Chromatogr. B Anal. Technol. Biomed. Life Sci..

[43] Bruheim P., Sletta H., Bibb M.J., White J., Levine D.W. (2002). High-yield actinorhodin production in fed-batch culture by a *Streptomyces lividans* strain overexpressing the pathway-specific activator gene actII-ORF4. J. Ind. Microbiol. Biotechnol..

[44] Pomraning K.R., Wei S., Karagiosis S.A., Kim Y.-M., Dohnalkova A.C., Arey B.W., Bredeweg E.L., Orr G., Metz T.O., Baker S.E. (2015). Comprehensive Metabolomic, Lipidomic and Microscopic Profiling of *Yarrowia lipolytica* during Lipid Accumulation Identifies Targets for Increased Lipogenesis. PLoS ONE.

[45] Pei G., Li X., Liu L., Liu J., Wang F., Chen L., Zhang W. (2017). De novo transcriptomic and metabolomic analysis of docsahexaenoic acid (DHA)-producing *Crypthecodinium cohnii* during fed-batch fermentation. Algal Res..

[46] Shen Y., Fatemeh T., Tang L., Cai Z. (2016). Quantitative metabolic network profiling of *Escherichia coli*: An. overview of analytical methods for measurement of intracellular metabolites. Trac-Trends Anal. Chem..

[47] Bligh E.G., Dyer W.J. (1959). A rapid method of total lipid extraction and purification. Can. J. Biochem. Physiol..

[48] Røst L.M., Shafaei A., Fuchino K., Bruheim P. (2020). Zwitterionic HILIC tandem mass spectrometry with isotope dilution for rapid, sensitive and robust quantification of pyridine nucleotides in biological extracts. J. Chromatogr. B Anal. Technol. Biomed. Life Sci..

[49] Xia J., Wishart D.S. (2016). Using MetaboAnalyst 3.0 for Comprehensive Metabolomics Data Analysis. Curr. Protoc. Bioinform..

[50] Minkler P.E., Kerner J., Ingalls S.T., Hoppel C.L. (2008). Novel isolation procedure for short-, medium-, and long-chain acyl-coenzyme A esters from tissue. Anal. Biochem..

